# Gene2Phenotype: A Database of Structured Human Monogenic Diseases and Pathomechanisms ^☆^

**DOI:** 10.1016/j.jmb.2026.169768

**Published:** 2026-03-26

**Authors:** Sarah E. Hunt, Diana Lemos, Seeta Ramaraju Pericherla, Olanrewaju Austine-Orimoloye, Elena Cibrian Uhalte, T. Michael Yates, Morad Ansari, Louise Thompson, Julia Foreman, T. Ian Simpson, James S. Ware, Caroline F. Wright, Mallory A. Freeberg, Helen V. Firth

**Affiliations:** 1 European Molecular Biology Laboratory, European Bioinformatics Institute, Wellcome Genome Campus, Hinxton, Cambridge CB10 1SD, the United Kingdom of Great Britain and Northern Ireland; 2 Biomedical Informatics Group, Institute for Machine Learning, School of Informatics, The University of Edinburgh, 10 Crichton Street, Edinburgh EH8 9AB, Scotland, the United Kingdom of Great Britain and Northern Ireland; 3 West of Scotland Clinical Genetics Service, Queen Elizabeth University Hospital, Glasgow, Queen, the United Kingdom of Great Britain and Northern Ireland; 4 South East Scotland Genetic Service, Western General Hospital, Edinburgh, the United Kingdom of Great Britain and Northern Ireland; 5 National Heart & Lung Institute, Imperial College London, London W12 0NN, the United Kingdom of Great Britain and Northern Ireland; 6 MRC Laboratory of Medical Sciences, Imperial College London, London W12 0HS, the United Kingdom of Great Britain and Northern Ireland; 7 Department of Clinical and Biomedical Sciences, Faculty of Health and Life Sciences, University of Exeter, Exeter EX1 2LU, the United Kingdom of Great Britain and Northern Ireland; 8 Wellcome Sanger Institute, Wellcome Genome Campus, Hinxton, Cambridge, the United Kingdom of Great Britain and Northern Ireland; 9 East Anglian Medical Genetics Service, Cambridge University Hospitals NHS Foundation Trust, Cambridge, the United Kingdom of Great Britain and Northern Ireland

**Keywords:** gene-disease associations, molecular disease mechanism, monogenic diseases, variant classification

## Abstract

To facilitate both disease research and personalised medicine, there is an urgent need for accessible, structured data models describing the molecular basis of genetically determined disease. Gene2Pheno-type is a database of expert-curated monogenic gene-disease associations, which was established in 2012 to enable efficient prioritisation of likely diagnostic genomic variants. Initially focused on developmental disorders, it has since been extended to support cardiac, eye, skeletal and skin disorders and germline cancer predisposition. We have redesigned and extended Gene2Phenotype, which now openly shares standardised, structured models of rare monogenic diseases, detailing genotype, molecular mechanism and associated phenotypes, curated from scientific literature. The updated platform, which includes a new API, enabling programmatic access, improves the findability, accessibility, interoperability and reusability of detailed rare monogenic disease association data. These data have the potential to accelerate disease research, clinical diagnosis, treatment selection and the development of novel therapies. Gene2Phenotype is available at https://www.ebi.ac.uk/gene2phenotype/.

## Introduction

This is a time of exciting potential in the study and management of genetically determined rare disease. Improvements in molecular biology techniques have enabled affordable, large-scale investigative and diagnostic genome sequencing [1]. Multiplexed assays of variant effect (MAVEs) can report on the cellular phenotype of all possible variants in an assayed region and could greatly accelerate diagnostic variant interpretation [2]. The development of tools to predict the impact of variants on gene function continues to be an active area of research, with tools utilising large genomic datasets and machine learning approaches being adopted in clinical settings [3]. Additionally, work is advancing on new treatments such as antisense oligonucleotide therapies [4], which could benefit those with rare disease. The molecular mechanisms underlying disease are complex [5] and understanding is crucial for designing novel therapies, so it is not sufficient to establish a gene-disease association and assume a loss of function model. Therefore, to help realise the potential of these advances to greatly enhance our understanding of rare disorders, well-structured, standardised models of the molecular basis of disease are needed. Specifically, detailed knowledge of pathomechanism and inheritance patterns is essential to improve specificity of clinico-genomic diagnosis, to ensure MAVE assays are informative for variant classification for a given disease, and to understand how to target novel treatments. Since >30% of monogenic disease-associated genes are associated with more than one condition [6], these detailed annotations are crucial for facilitating robust variant interpretation.

The literature is a rich source of reports on gene-disease association, but information must be evaluated, structured and standardised to enable its use in bioinformatics pipelines. The Gene2Phenotype (G2P, https://www.ebi.ac.uk/gene2phenotype/) database is a FAIR (Findable, Accessible, Interoperable and Reusable) resource which addresses this challenge by openly sharing detailed, expert-curated, clinician-reviewed models of monogenic rare diseases. It was established in 2012 to enable efficient, flexible and scalable filtering of genomic variants in the diagnosis of genetically heterogeneous clinical presentations in the Deciphering Developmental Disorders study [7]. A genotype filtering tool built over the Ensembl Variant Effect Predictor (VEP) [8] utilises G2P data to identify likely causative variants from exome or genome sequencing data. Many clinical diagnostic laboratories now employ this method, utilising virtual gene panels – lists of genes known to be associated with a type of disorder – as an efficient approach to variant prioritisation. When a patient’s sample is sent for testing, whole exome or genome sequencing is performed but only variants in genes from the panel(s) relevant to their phenotype are considered. To facilitate such use, G2P data is grouped by domain experts into virtual gene panels.

The G2P data model has been extended to capture more detailed molecular mechanism information and the platform reimplemented to enable more intuitive data discovery and exploration. G2P now enables easy access to standardised, detailed models of gene disease association with a focus on molecular mechanism. This helps unlock the potential of recent technological advances to accelerate rare disease research, diagnosis and management.

## Results

The core unit of the G2P database is the G2P record, which describes the molecular basis of a genetically determined disorder. G2P records are curated by clinicians, clinical scientists and scientific curators, and each disease area is overseen by a physician with expertise in that domain. In response to the urgent clinical need for more in-depth understanding of pathomechanism, the curation process was recently updated to capture richer annotation, including in-depth molecular mechanism information, whilst retaining a scalable standardised data model [9]. Briefly, peer reviewed publications describing the results of genome-sequencing patients with specific disorders are identified, and information on the number of patients studied, their phenotypes, genotypes and evidence supporting likely mechanism are extracted into a standard format. Disease mechanism is inferred based on the action of the variants observed in affected individuals and the functional evidence reported (e.g. ‘RARB-related microphthalmia syndromic’ is annotated ‘gain of function’ based on reports that missense variants observed in patients increase transcriptional responsiveness to retinoic acid ligands, disrupting normal development). Evidence supporting both the association and the mechanism is evaluated, and a confidence level and disease mechanism are agreed within the expert curation team. We have developed a new curation tool which facilitates efficient capture of this data. It ensures robust use of community ontologies, displays information from other resources to guide decision making and performs quality checking to reduce data entry errors (Supplementary Methods). The tool makes the curation process faster and easier to standardise between different curators. The G2P website has been redesigned to facilitate more intuitive data discovery and dissemination. To maximise the impact of G2P curation, our data management strategy follows FAIR principles [10]. Specifically, G2P data is described using domain-relevant community standards and ontologies to maximise findability and interoperability; an improved browser and API aid accessibility and links to the publications records are built from are reported to support informed reuse.

### Data model

**Figure 1:**
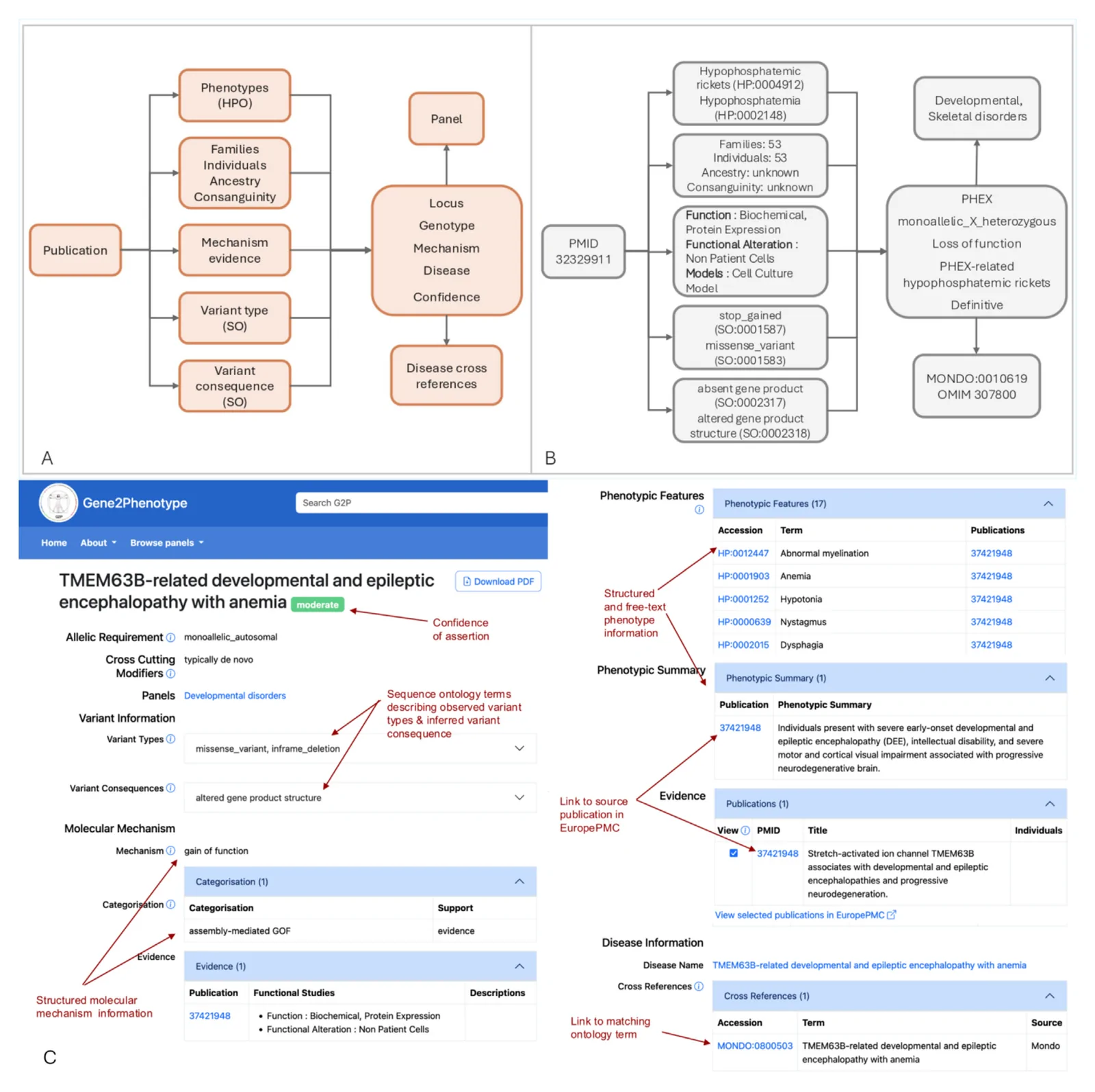
A – The structured G2P record showing ontology terms used; B – Example record (https://www.ebi.ac.uk/gene2phenotype/lgd/G2P03769) illustrating how the model is populated; C – An example of the G2P record page displaying the curated data and links to source publications. (https://www.ebi.ac.uk/gene2phenotype/lgd/G2P03520).

The G2P data model captures curated gene-disease associations with supporting evidence (Figure 1). It was designed as a modular architecture to enable the addition of new data types and relationships without requiring changes to core components. The core of the model is a unique Locus-Genotype-Mechanism-Disease-Evidence (LGMDE) thread [11].

- The **locus** is a genomic feature or region. Currently G2P holds data only for genes, but the model is flexible to support records based on other loci, such as single variants or regulatory regions.
- The **genotype** records the allelic requirement, such as whether the condition is observed in homozygous (biallelic) or heterozygous (monoallelic) individuals. Additional inheritance qualifiers are supported to record, for example, disorders with incomplete penetrance or those typically observed de novo.
- The disease **mechanism** is reported as a high-level term (one of: loss of function (LOF), gain of function, dominant negative, undetermined non-loss-of-function) with a more specific categorisation (e.g. ‘interaction-disrupting LOF’) where possible, using predefined lists, derived from Backwell and Marsh [5]. The ‘undetermined non-loss-of-function’ term is used for associations where there is convincing evidence that mechanism is not loss of function, but where there is insufficient evidence to distinguish between dominant-negative and gain-of-function effects. As this is not a definitive mechanism, we monitor the literature for further evidence. (An example is ‘HK1-related neurodevelopmental disorder with visual defects and brain anomalies’, where missense variants were observed in patients and biochemical assays performed on their cells showed there was no decrease in the activity of the gene product. Additionally, biallelic loss of function in HK1 is associated with a different disorder). To aid understanding of pathomechanism, the types of variants observed (e.g. missense, stop gained) are recorded, and a likely variant consequence (e.g.altered gene product structure, absent gene product) is reported using Sequence Ontology (SO) terms defined by the Gene Curation Coalition (GenCC) [12].
- **Diseases** are named using a standardised gene-specific dyadic approach [13] and observed pheno-types are reported as Human Phenotype Ontology (HPO) [14] terms.
- Publications used to build the record are reported as **evidence**. Important information, including the number of affected individuals and families reported, their ancestries and types of assays supporting the recorded disease mechanism are extracted and reported as structured attributes. This information, as well as the number of publications supporting the association and the length of time it has been upheld, are used to determine the confidence of the association which is reported using terms harmonised within the Gene Curation Coalition (e.g. strong, limited) [15].

To support accessibility, the LGMDE thread is presented as a G2P record, which is assigned a unique and persistent identifier, the G2P ID. This ID can be used to extract all current information programmatically via the G2P API or to create a predictable URL, enabling direct linking to the specific record page on the G2P website. Occasionally records are merged, in response to more knowledge becoming available about a condition. When this happens, the G2P ID remains searchable, returning the record into which it was merged. To aid interoperability, curators annotate G2P records with equivalent Mondo Disease Ontology (Mondo) [16] and OMIM [6] identifiers where possible.

### Data available

There are currently 3828 G2P records organised into 7 overlapping panels (Table 1).

- 85% ⟶ have a confidence of ‘definitive’, ‘strong’ or ‘moderate’ and are used in clinical reporting.
- 65% ⟶ have HPO terms.
- 69% ⟶ have a mechanism listed.
- 80% ⟶ are linked to an OMIM identifier.
- 89% ⟶ are linked to a Mondo term.

Records with little evidence (‘limited’ confidence) are most likely to be missing these attributes.

### Website

The G2P website provides a flexible search interface, allowing free text queries over genes, diseases, and phenotypes as well as lookup by G2P ID. To improve findability, searching can be performed using:

- gene identifiers from HGNC, Ensembl and OMIM
- disease descriptions and identifiers from OMIM and Mondo
- phenotype descriptions and accessions from HPO

**Figure 2:**
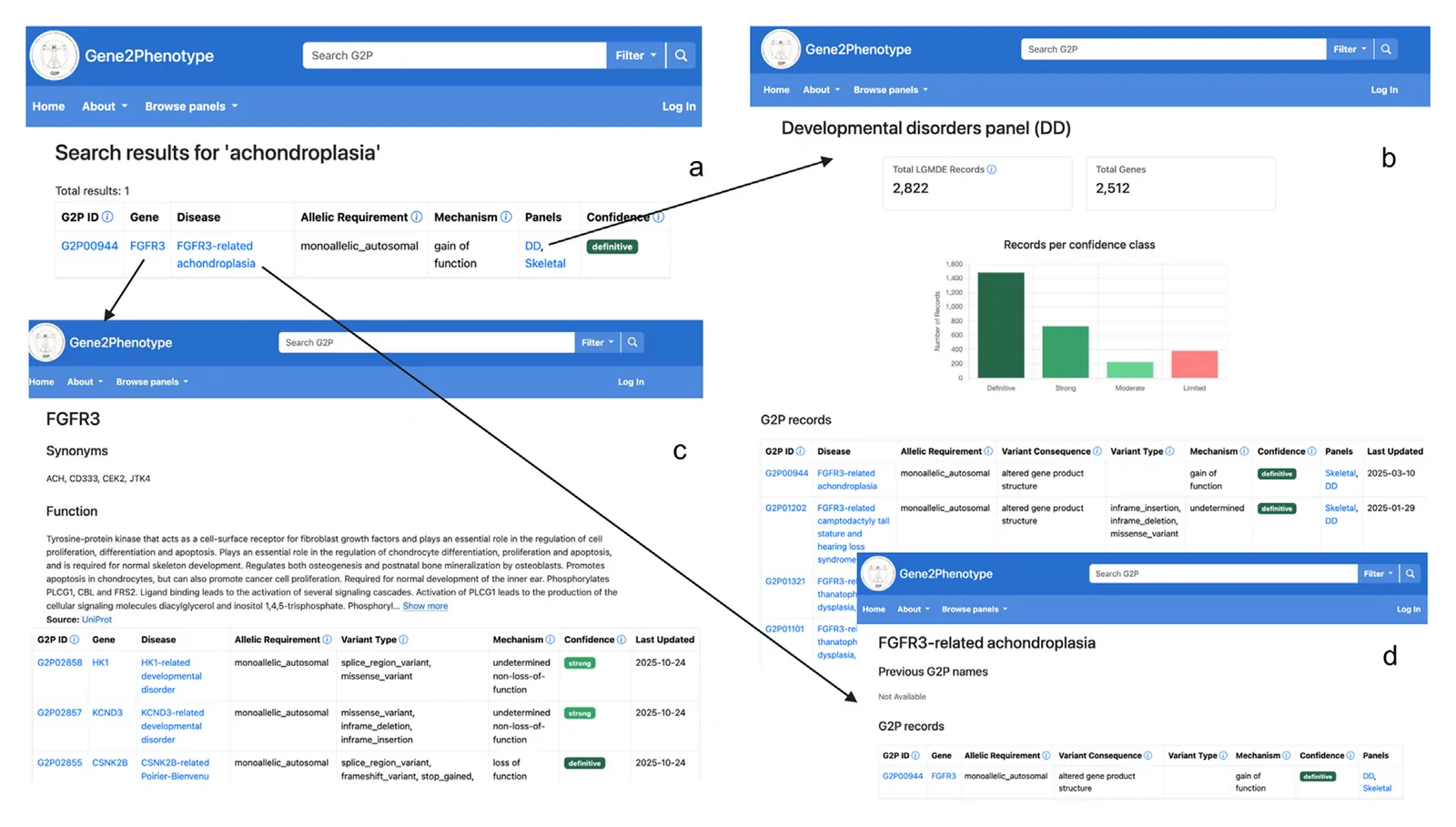
Example pages from the G2P website. (a) Search results with links to views focused on; (b) the virtual gene panel; (c) the gene; (d) the disease.

Searches can be performed over the whole dataset or be faceted to specific data types (e.g. gene, disease, phenotype) and/or panels (e.g. DD, Cancer, Cardiac). Search results include a summary of matching records (Figure 2a), with links to pages focused on relevant G2P records, the genes, diseases and the panels.

Panel pages (Figure 2b) display a graphical view of the number of records available, broken down by confidence rating and a summary of the most recently updated records, as a prompt to remind users to update their local copy. A link is available to download full panel information. Gene pages (Figure 2c) show a summary of the G2P records available for that gene along with protein function information from UniProt [17] and links to key external resources, enabling easy access to further information. Disease pages show summaries of linked G2P records with cross-references to OMIM and Mondo and any previous names the disease was known by in G2P.

The G2P record page (Figure 1c) is the focal point of the website bringing together curated information and aggregated reference data in a single interface.

The page displays structured knowledge on the locus, genotype, mechanism and disease, with indication of evidence strength, and provides links to the publications used to create the record, enabling convenient examination of the source data.

### API and data downloads

Recognising the community’s need for a simple way to integrate gene-disease information into resources and workflows, we developed a public RESTful API to enable programmatic access to G2P data. It supports search functionality as described above, reporting when information is available for genes and diseases identified by commonly used identifiers. More detailed information is available via the ‘summary’ endpoints which report key information for a specific gene symbol or a Mondo or OMIM disease accession. Full details of a G2P record can be retrieved using the G2P ID; this includes the core information of gene, allelic requirement, variant consequence mechanism and the publications relating to the record as well as phenotypes, mechanism categorisation, functional evidence and variant types where available (Supplementary Figures 1–7). Data can be downloaded in bulk by querying the API using a panel identifier or using ‘All’ as the global panel name.

In addition to API access, the latest data can be exported via the website. To enable archiving of the exact information available at the time a decision was made, a single entry can be exported as a PDF file from record pages. As G2P is continuously updated when new evidence emerges, it is recommended to use the live site for other purposes, especially when patient data is reanalysed. A link in the PDF file provides direct access to the latest information to support this. To enable easy upload into other databases for use in analysis workflows, key details on all records can be downloaded by panel from the Download or Panel pages. To aid reproducibility of analysis, monthly data freezes are made available on the G2P FTP site and maintained as an archive. A comma-separated file with one record per line is provided, to be compatible with Excel for use by clinicians and simple parsing for use in AI algorithms or analysis pipelines.

### Diagnostic variant filtering

To facilitate efficient and flexible filtering of patient genotypes and aid the identification of plausibly diagnostic genomic variants, we developed the VEP-G2P extension [11] to Ensembl VEP (Supplementary Figure 8). Briefly, proband genotypes are filtered to identify those which are rare or not observed in population cohorts and are predicted to disrupt a known disease-related gene. The tool compares the patient’s genotype to the recorded allelic requirement and reports transcripts with at least one heterozygous genotype for monoallelic conditions and either one homozygous genotype or two heterozygous genotypes for biallelic conditions. Reporting genes with two heterozygous genotypes will include false positives when the alleles are in cis, but it is preferable to over report until the production of phased data is common and the filter can be updated.

VEP-G2P was used to evaluate the developmental disorders panel with 4293 trios and found to have a sensitivity/precision of 97.3%/33% for de novo and 81.6%/22.7% for inherited pathogenic genotypes [9] while similar benchmarking of the Eye panel in the diagnosis of 1234 individuals with eye disorders gave an increase in precision of 35% over routine methods [19]. Evaluation of Cardiac panel in the prioritisation of 285 variants resulted in a sensitivity of 98.6% [18]. All studies showed a reduction in the number of variants the clinical scientist must evaluate, demonstrating the value of G2P’s structured data in a first-tier variant filter and facilitator of routine reanalysis.

Since our last report [11], we have further refined the filtering step to utilise newly curated information, optionally matching the predicted molecular consequence of the patient genotype to the observed variant consequence reported in G2P. Strict and tolerant matching is available. For example, if a G2P record has a variant consequence of ‘absent gene product’, any variants with Ensembl VEP predicted consequence of ‘splice_donor_variant’, ‘start_lost’, ‘frameshift_variant’ or ‘stop_gained’ will be reported in strict mode. In tolerant mode, ‘splice_ acceptor_variant’ will also be reported.

## Discussion

G2P was initially created to capture information on developmental disorders but has been generalised to other disease areas. The level of information available in published reports of disease association has increased since G2P was designed and understanding of the complexity of the molecular mechanism of disease has increased. Importantly, the G2P data model has been extended to capture detailed, standardised information on molecular disease mechanism. This can improve efficiency in diagnostic variant filtering, enabling more precise matching while saving time, and supports precision medicine. The G2P infrastructure redesign enables access for a range of uses including exploration and discovery as well as integration into production workflows. It introduces major improvements to support higher data quality and greater accessibility, in particular improved curator guidance and error checking provided by the updated curation interface, and the REST API for programmatic access.

### Collaboration with similar resources

Multiple international groups curate gene-disease associations, each with different priorities and therefore varying approaches to the level of detail captured, data formatting and dissemination. This approach is beneficial as groups can more rapidly trial and adopt different processes, and comparison of methods and results across groups can be performed to raise curation quality, benefiting the whole community. As the curation of gene disease associations is a time-consuming endeavour however, it is desirable to reduce redundancy of effort. We therefore collaborate to maximise the benefit of our efforts, including working with the Mondo team to create names and definitions for the novel diseases we curate to support consistency of disease naming in the community.

G2P is a founding member of the GenCC, which was established to harmonise gene level resources by agreeing standards for data description and association confidence reporting. Members also submit basic information on gene-disease associations to the GenCC database, which holds information on 5668 genes to date, and meet to discuss and resolve any disagreements on confidence level, improving data quality across the community. 3468 G2P records have been submitted to GenCC to date, making it the largest contributor of confidence-rated gene disease association reports with supporting publications among the open-access resources (Supplementary Table 1).

The detailed curation of the latest developmental disorder reports is prioritised in G2P, to drive up diagnostic rates in this complex area in the UK National Health Service (NHS). Other disorders are supported where clinical experts are available to contribute and ensure data quality. The G2P DD panel is used directly in NHS Scotland and G2P records are imported into PanelApp for use in NHS labs in England and Wales as part of the Paediatric Disorders gene panel (R27) in the National Genomic Test Directory. The G2P Cardiac panel was created in collaboration with the ClinGen Heritable Cardiovascular Disorders Gene Curation Expert Panel; pre-existing records were re-curated to add the variant consequence information required in the G2P data model. We continue to deepen our collaborations with other teams to share effort and increase the coverage of further disease areas in our respective resources.

Some groups also provide online resources enabling open browsing or download of data. OMIM was the first project to capture genedisease data and now holds extensive descriptive reports on 5051 genes with mutations causing diseases, traits and susceptibilities to cancer and complex diseases. The reader can browse free-text descriptions derived from the literature on gene- and phenotype-specific pages on the OMIM website. The ClinGen Clinical Genome Resource [20] provides curated information for 3461 genes, 2838 of which have disease associations reported using a robust standardised assessment of the available evidence. Lists of gene-disease associations, with standardised confidence ratings can be downloaded in bulk and details of the evidence available for a gene can be browsed on the ClinGen website. The Genomics England PanelApp resource [21] prioritises evaluating evidence for a disease association sufficient to enable the addition of a gene to diagnostic screening panels over more detailed curation and makes data for 5960 genes available through a web browser and API. G2P’s unique value lies in the level of detail captured and reported in a consistent structured format – specifically the variant consequence and mechanism information – and in providing open, easy access to all data for 3158 genes via an API, bulk download as well as a browsable website.

### Use in other resources and processes

In accordance with the initial mission to improve diagnostic rates for those with genetically heterogeneous monogenic disorders, G2P data is routinely used to facilitate diagnostic filtering of short variants and is recommended for use in structural variant interpretation in the Best Practice Guidelines for Variant Classification in Rare Disease produced by the Association for Clinical Genomics Service in the United Kingdom. G2P data is integrated into other resources including the OpenTargets Platform [22], which supports drug target identification and prioritisation, and the Ensembl [23] and UCSC [24] genome browsers, leading platforms used by the global research community for genomics data discovery and visualisation. G2P has also been used in the development of AI tools [25,26] and is recommended for use in checking that MAVE assays are relevant to the disease mechanism before use in clinical variant interpretation [27].

### Limitations and future plans

Limitations of the G2P database arise from the time-consuming nature of manual curation and finite expert-curation capacity. G2P targets multiple disease areas, with a particular focus on developmental disorders, but does not have full coverage of all monogenic diseases. Also, we have recently extended curation protocols to cover additional attributes, but due to the need to balance the curation of new conditions with review and upgrade of existing records, many older records have not yet been reviewed and updated to the new standard. For example, a third of entries do not have HPO terms curated and a similar number have no mechanism information, but we expect there is sufficient evidence available to deduce mechanism for a large proportion of these when they can be re-examined.

We are engaging with further domain experts to extend G2P to new disease areas and are strengthening collaborations with other curation projects to minimise redundancy of effort. To accelerate the curation process, we are working to mine information from the literature by natural language processing methods and use it to seed or enhance G2P records, continuing previously reported work [28]. An early goal is to improve coverage of phenotype terms in G2P by this approach. We have developed a machine-learning pipeline to identify publications relevant to G2P gene-disease associations [29] and are using it to build an automated literature surveillance system, which will flag records for review when new data is available. This will shift the curator role to focus on evaluation of the data presented, reducing the manual identification of relevant information and data entry tasks, utilising time more efficiently. To ensure clarity for down-stream users, mined data will be displayed along-side curated information on the website, data downloads and API responses, but will be clearly marked if not yet reviewed. This will maintain the current record quality while reducing manual effort and provide information for process tuning. Other efforts are ongoing in this area [30] providing opportunities for comparison and further method refinement.

## Conclusions

In providing open, easy access to detailed, structured gene-disease data models, with a focus on molecular pathomechanism, G2P has the potential to facilitate research into monogenic disease; accelerate diagnostic variant prioritisation, aid robust clinico-genomic diagnosis and improved management; support tool development and aid the development of therapies and identification of novel drug targets.

## Materials and Methods

### Design and implementation

The new G2P platform was designed in collaboration with clinicians and clinical scientists and genomics researchers to ensure usability. The API is implemented in Python and the website in JavaScript, using well maintained frameworks (Supplementary Methods – Implementation) for sustainability.

### Data curation and validation

As curation is a laborious process, requiring the extraction of information from unstructured text, mapping to standardised terms and careful data entry, we developed an improved web-based curation interface to guide curators through a standardised protocol. The tool enables selection of terms from controlled vocabularies, performs quality assurance checks (e.g. the allelic requirement must be compatible with the genomic location of the gene; records cannot be assigned high confidence levels without the appropriate number of publications being recorded) and prevents the addition of erroneous duplicates (Supplementary Methods – Data curation and validation), reducing the time and effort needed while improving consistency.

G2P records are curated by teams composed of clinical scientists, practising clinicians and scientific curators. Team members have different data publishing and update privileges according to experience level. Individuals curate papers and create draft G2P records, which capture the reported evidence and the curators’ recommendations for confidence level, mechanism and disease name in a structured format. Draft records are presented in curation team meetings, evidence reviewed and aspects such as mechanism, confidence and disease name are discussed and agreed.

## Supplementary Material

Supplementary material to this article can be found online at https://doi.org/10.1016/j.jmb.2026.169768.

Supplementary information

## Figures and Tables

**Table 1:** Records available in G2P as of 2026-01-06.

Panel	Cancer	Cardiac	DD	Ear	Eye	Skeletal	Skin
Records	125	128	2839	98	790	345	608
Genes	113	112	2519	88	664	296	485
Confidence definitive	97 (77.6%)	69 (53.9%)	1479 (52.0%)	72 (73.4%)	544 (68.8%)	291 (4.3%)	441 (72.5%)
Confidence strong	9 (7.2%)	5 (3.90%)	731 (25.7 %)	16 (16.3%)	132 (16.7%)	33 (9.56%)	64 (10.5%)
Confidence moderate	13(10.4%)	8 (6.25%)	240 (8.45%)	5 (5.10%)	6 (0.75%)	6 (1.73%)	41 (6.74%)
Confidence limited	6 (4.8%)	46 (35.9%)	389 (13.7%)	5 (5.10%)	108 (13.6%)	15 (4.34%)	62 (10.1%)
HPO available	114 (91.2%)	14 (10.9%)	1753 (61.7%)	88 (89.7%)	664 (84.0%)	296 (85.7%)	552 (90.7%)
OMIM available	83 (66.4%)	14 (10.9%)	2459 (86.6%)	90 (91.8%)	663 (83.9%)	338 (97.9%)	499 (82.0%)
Mondo available	99 (79.2%)	128 (100%)	2574 (90.6%)	88 (89.7%)	720 (91.1%)	331 (95.9%)	523 (86.0%)
Mechanism available	113 (90.4%)	34 (26.5%)	2086 (73.4%)	76 (77.5%)	557 (70.5%)	282 (81.7%)	449 (73.8%)
Variants available	14 (11.2%)	82 (64.0%)	898 (31.6%)	23 (23.4%)	201 (25.4%)	77 (22.3%)	79 (12.9%)

## Data Availability

G2P data is freely available via the G2P website: https://www.ebi.ac.uk/gene2phenotype/.
