# Supplementary material for: Gene2Phenotype: A Database of Structured Human Monogenic Diseases and Pathomechanisms
Source: J Mol Biol. Author manuscript; Available in PMC 2026 Nov 11. (PMC7619723; doi:10.1016/j.jmb.2026.169768)
Supplement: Supplementary file 1 [file EMS222201-supplement-Supplementary_information.pdf]

Supplementary information

Supplementary information **1**

- Supplementary results 1
  - API data access 1
  - Diagnostic variant filtering 7
  - Data comparison 8
- Supplementary Methods 9
  - Implementation 9
  - Curation and data validation 10
- Additional references 12

Supplementary results

API data access

The new G2P API enables more flexible access to G2P data than the original system which supported only one query: the download of full data for a panel. This option is available in the new API along with a search endpoint which returns summary information for a gene symbol, a disease, (by description, Mondo or OMIM identifier) or a phenotype (by description or HPO term). Wildcard searches are performed when descriptions are entered and the endpoint supports limiting the entities returned (to genes, phenotypes or diseases) and filtering of results by panel, mechanism and variant consequence. A further endpoint supports look up using a G2P identifier to return all details for a record. Full documentation for the new API is available from https://www.ebi.ac.uk/gene2phenotype/g2p-api-info.

The new search endpoint (Supplementary Figure 1) returns a summary of the key information for all matching G2P records, including the G2P identifier.

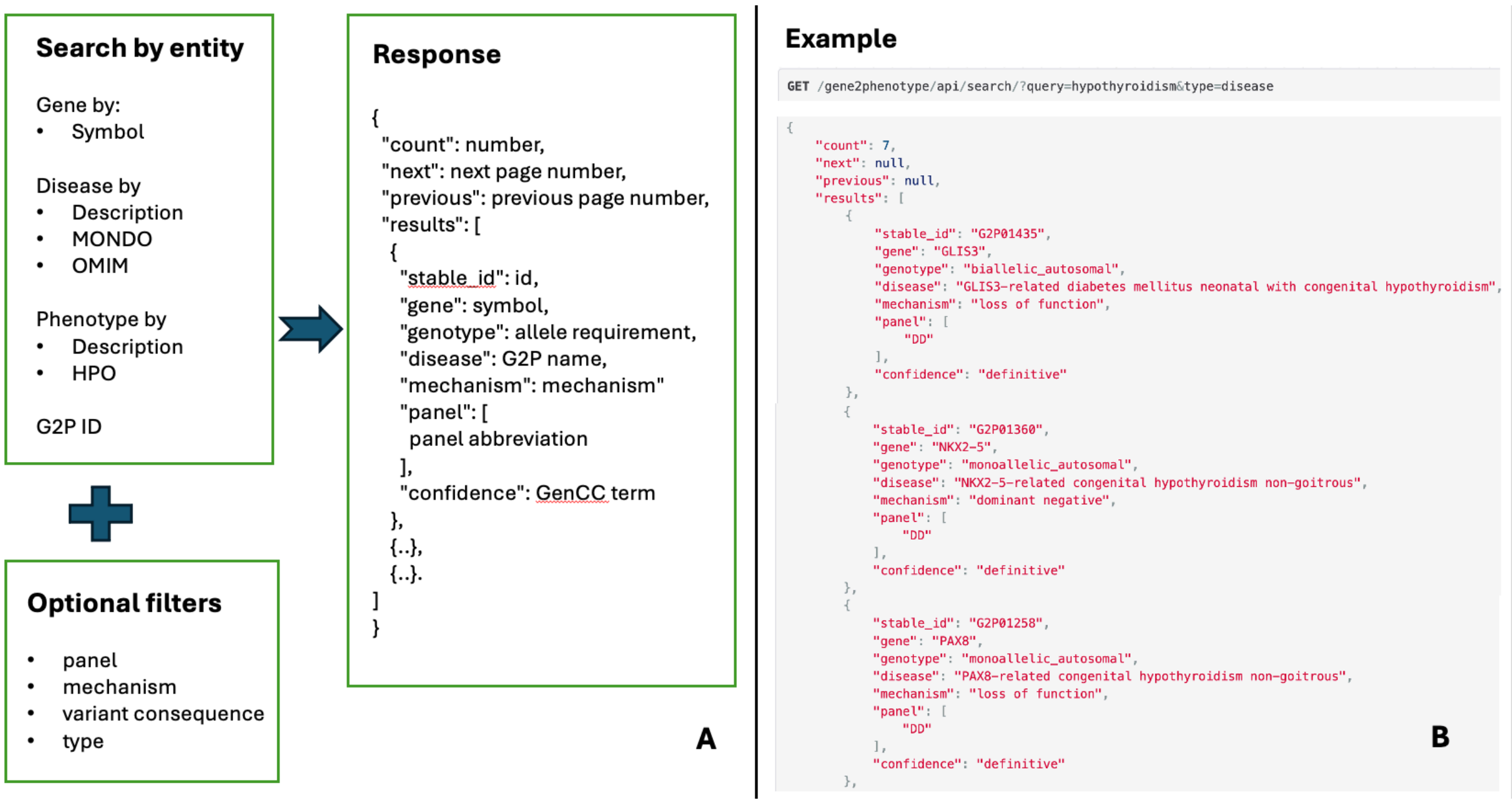

Supplementary Figure 1: Search options in the new G2P API. (A) Search parameters and response structure. (B) Example query using a text string 'hypothyroidism' and filtering to search only disease names. This returns summary reports for 7 G2P entries with 'hypothyroidism' in the disease name. Searching filtering by type 'phenotype' would currently return 45 entries where hypothyroidism is listed as a phenotype associated with the disease.

The G2P identifier can be used to return all information held for a G2P record.The response to the query "https://www.ebi.ac.uk/gene2phenotype/api/lgd/G2P03734" is detailed in Supplementary Figures 2-7

```
{
  "locus": {
    "gene_symbol": "UBR5",
    "sequence": "8",
    "start": 102252273,
    "end": 102412759,
    "strand": -1,
    "reference": "grch38",
    "ids": {
      "HGNC": "HGNC:16806",
      "Ensembl": "ENSG00000104517",
      "OMIM": "608413"
    },
    "synonyms": [
      "DD5",
      "EDD",
      "EDD1",
      "HYD",
      "KIAA0896"
    ]
  },
  "stable_id": "G2P03734",
  "genotype": "monoallelic_autosomal",
```


Genomic location of the gene

Other common gene identifiers

Previous gene symbols

Allelic requirement

Supplementary Figure 2. Section of REST API response detailing the locus described in G2P03734, with G2P identifier and genotype.


```
 "variant_consequence": [
   {
     "variant_consequence": "altered gene product structure",
     "accession": "SO:0002318",
     "support": "inferred",
     "publication": null
   },
   {
     "variant_consequence": "decreased gene product level",
     "accession": "SO:0002316",
     "support": "inferred",
     "publication": null
   }
 ],
 "molecular_mechanism": {
   "mechanism": "loss of function",
   "mechanism_support": "evidence",
   "synopsis": [
     {
       "synopsis": "loss of activity LOF",
       "support": "evidence"
     }
   ],
   "evidence": {
     "39721588": {
       "functional_studies": {
         "Function": [
           "Biochemical"
         ],
         "Functional Alteration": [
           "Non Patient Cells"
         ],
         "Models": [
           "Non-Human Model Organism",
           "Cell Culture Model"
         ]
       },
       "descriptions": []
     }
   }
 },
```


Variant consequence inferred from observed variant types

High level mechanism

More specific mechanism categorisation

Publication providing evidence

Type of evidence reported

Supplementary Figure 3. Section of REST API response detailing the variant consequence and mechanism information in G2P03734.

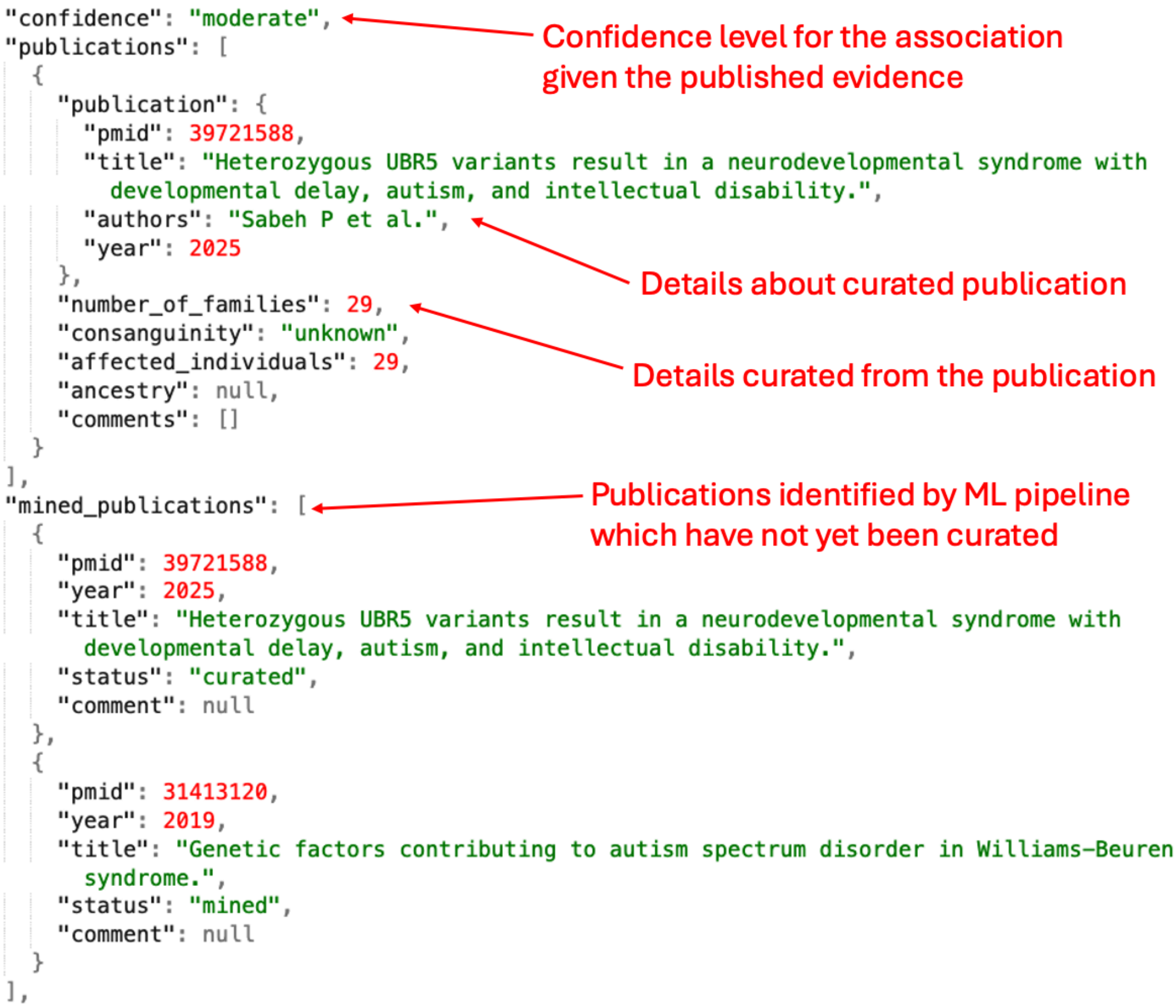

Supplementary Figure 4. Section of REST API response detailing the evidence supporting the gene disease association described in G2P03734 and the assigned confidence level.

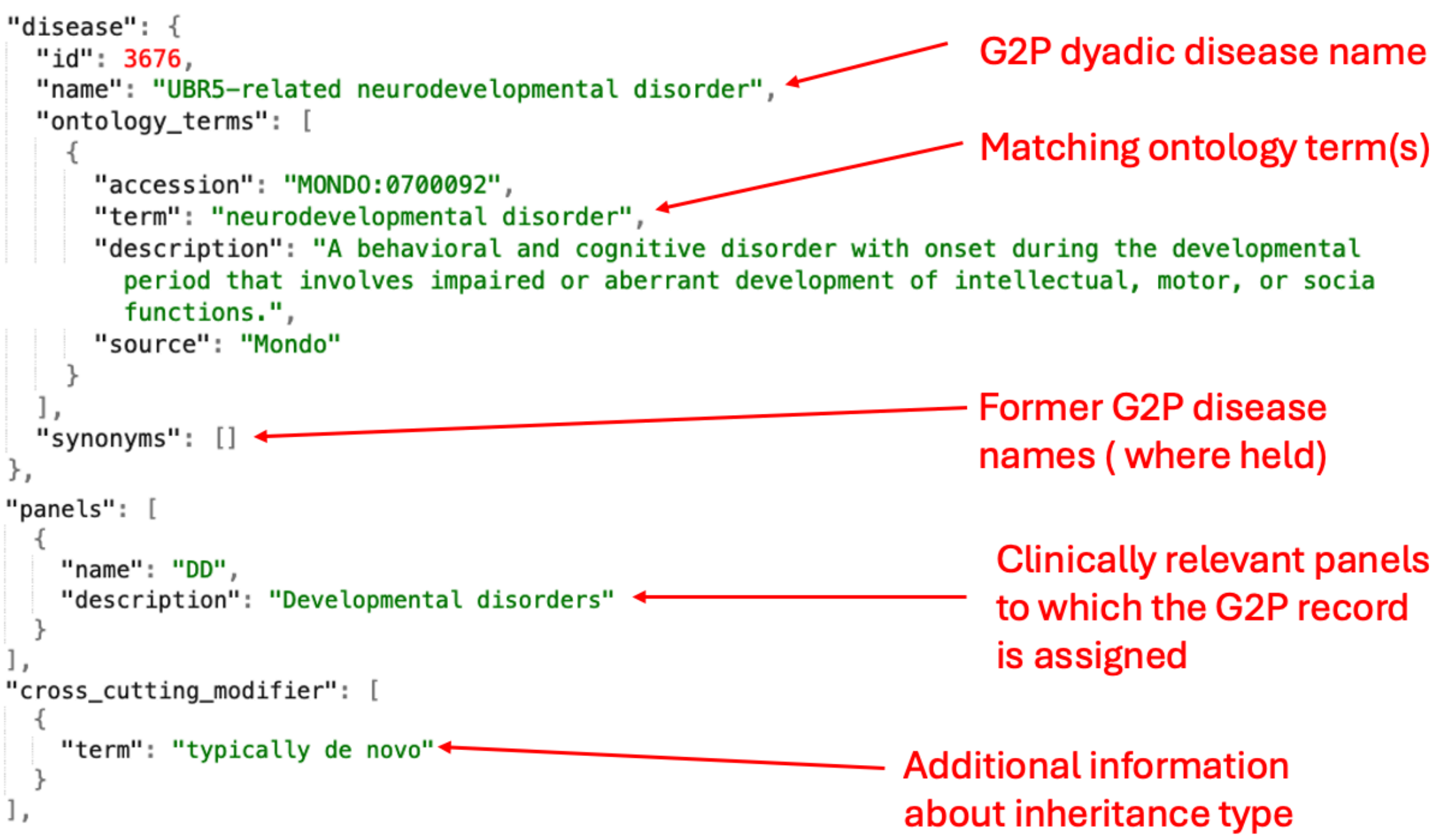

Supplementary Figure 5. Section of REST API response detailing the disease, panel and inheritance modifier described in G2P03734.

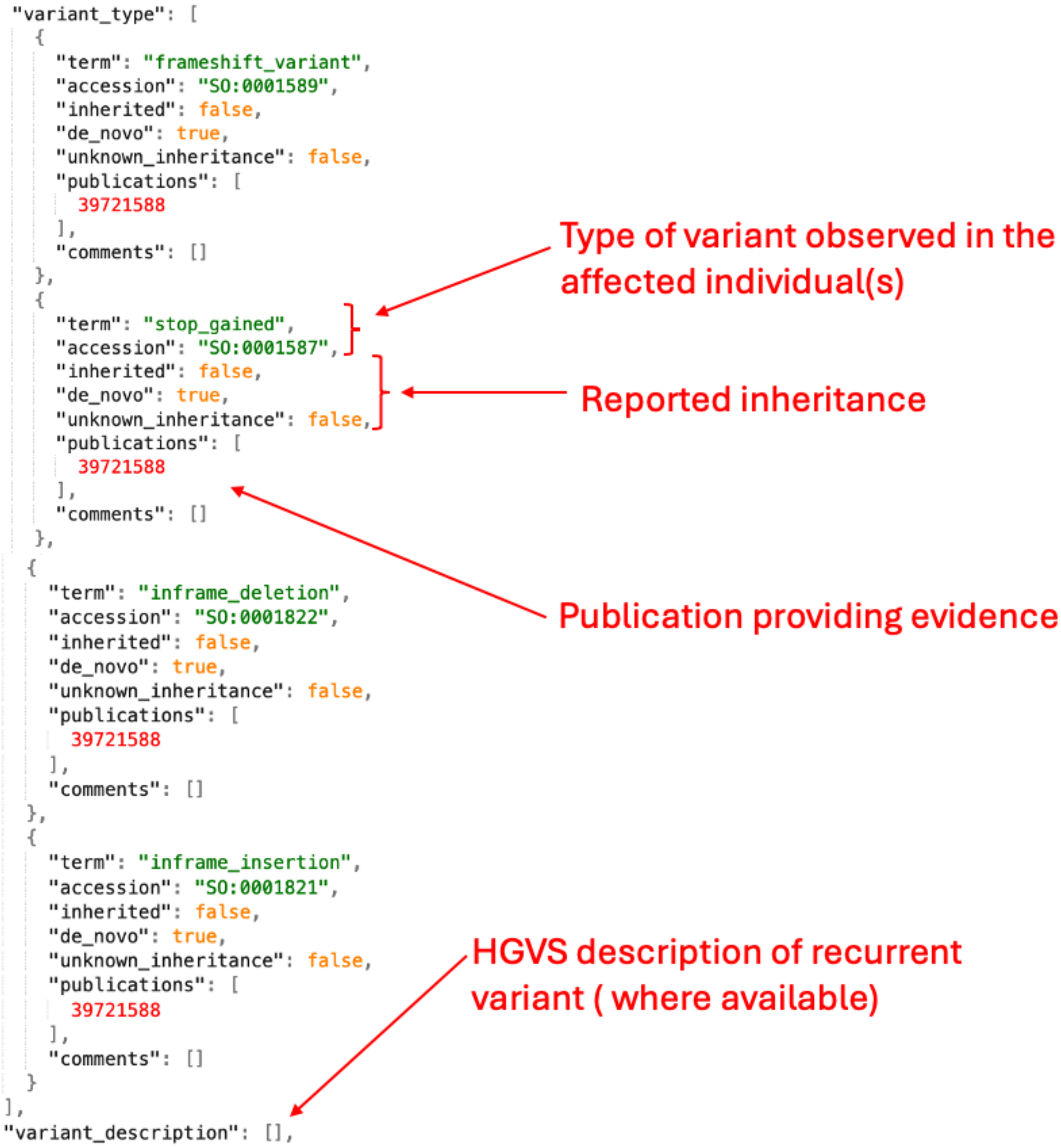

Supplementary Figure 6. Section of REST API response detailing the variant types reported in affected individuals in G2P03734.

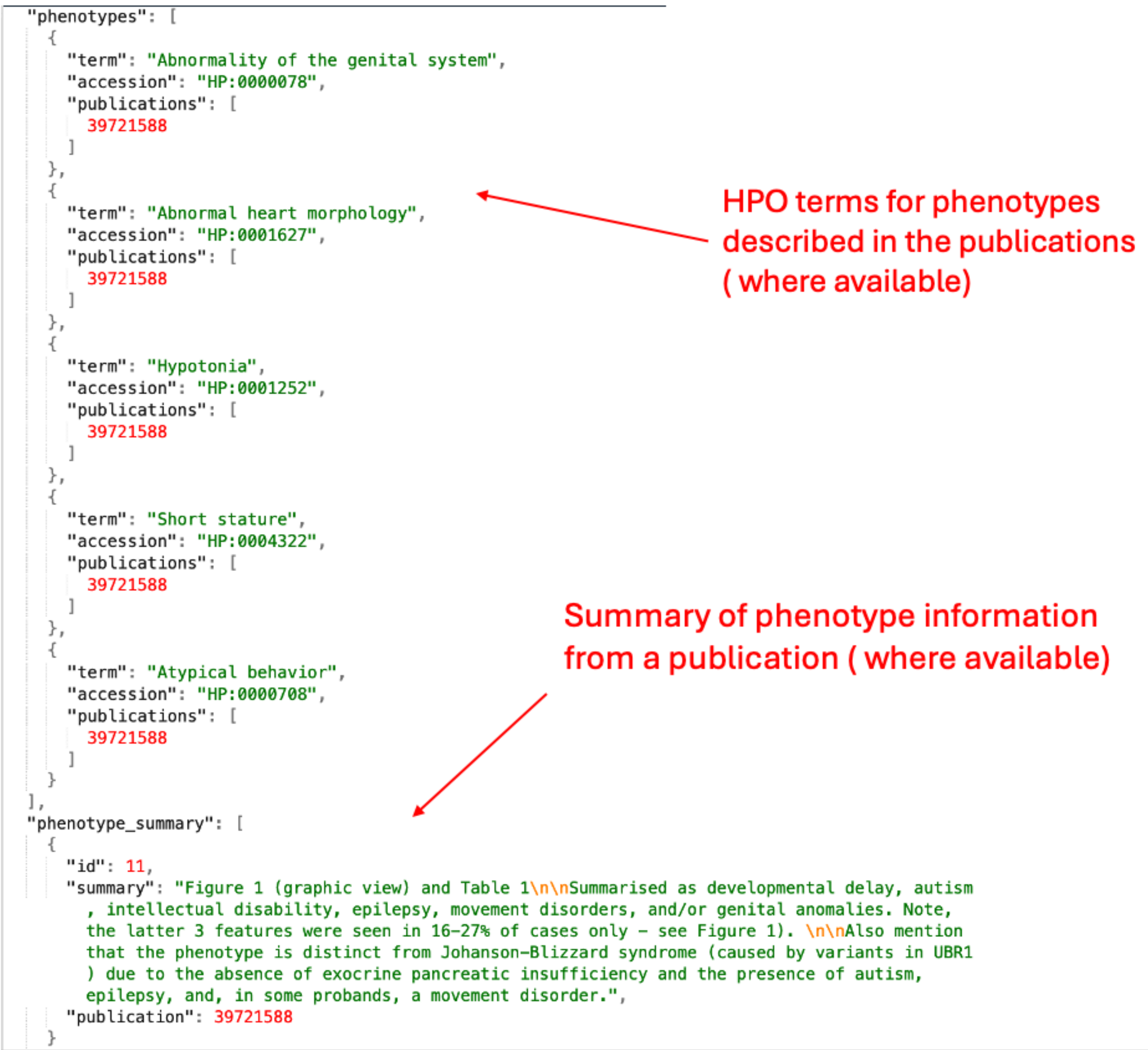

Supplementary Figure 7. Section of REST API response detailing the phenotype information extracted for G2P03734. Note - the image does not include all HPO terms for brevity.

Diagnostic variant filtering

The use of G2P data in diagnostic variant filtering is outlined in Supplementary Figure 8 and fully described in previous reports [11, 18,19]. Briefly, the proband's genotypes are mapped to genes and annotated with predicted molecular consequence by Ensembl VEP. The Ensembl VEP G2P plugin identifies variants overlapping genes known to be associated with the proband's phenotype and matches molecular consequence to disease mechanism. Variants more common in gnomAD than the disorder is in the population are filtered out then the proband genotype for a gene is filtered to match the recorded allelic requirement of the disorder. The genes for which variants pass these filters and genes in which the proband has known recurrent variants (entered as a 'Variant inclusion list') are reported for expert clinical review.

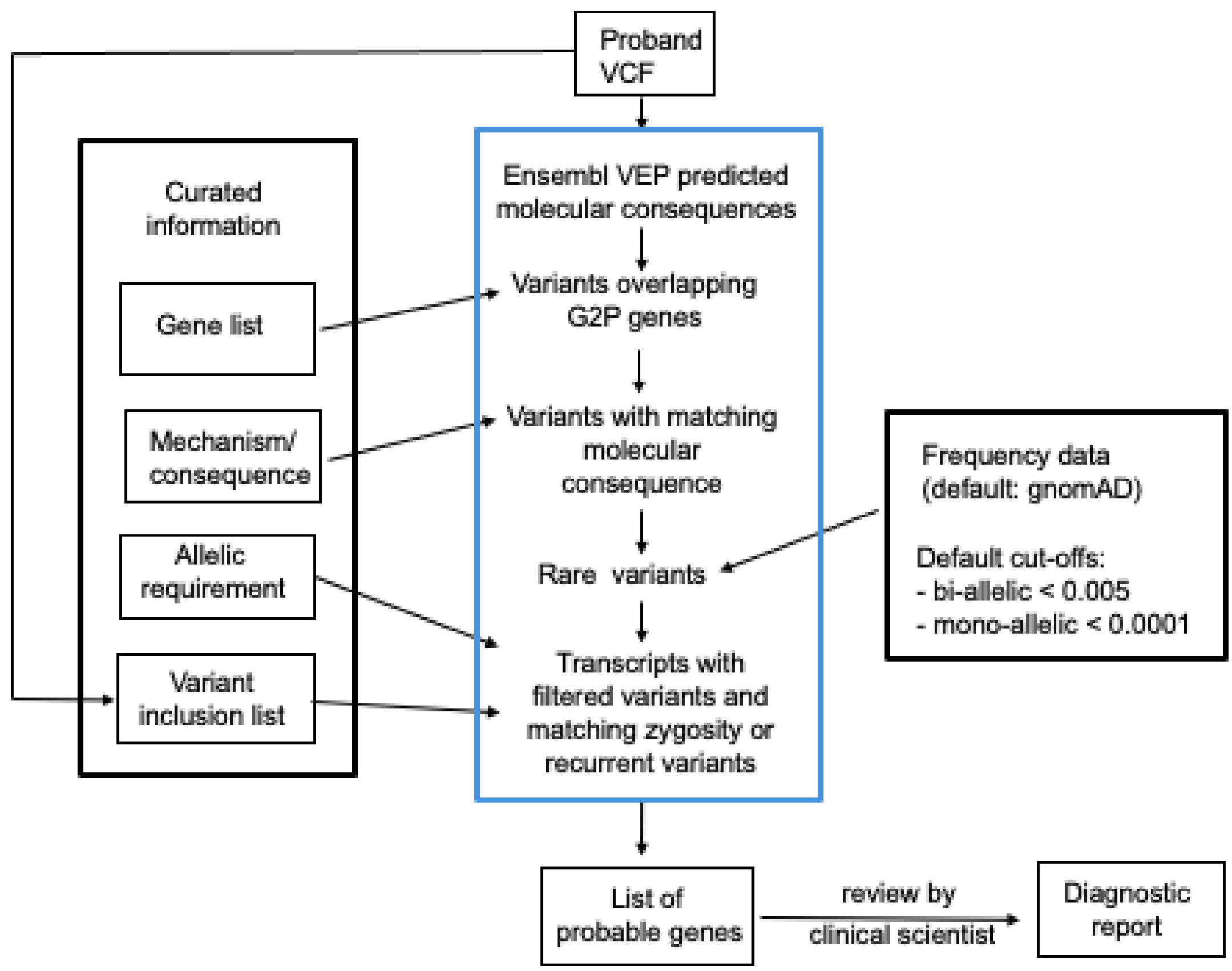

Supplementary Figure 8. Diagnostic variant filtering process using structured G2P data.

Data comparison

Multiple groups curate gene-disease associations, each with different priorities and therefore varying approaches to the level of detail captured, data formatting and dissemination. While OMIM and ClinGen often report likely disease mechanism in free text comments, G2P is currently the only resource reporting structured mechanism information for improved reusability.

Many curation groups submit summary information to GenCC which enables checking and refinement of approaches across the community. Supplementary Figure 9 shows the number of genes for which at least one record was submitted to GenCC by four of the open gene curation resources (ClinGen, G2P, Genomics England PanelApp and Orphanet). This does not reflect the latest data in the resources but does indicate level of overlap.

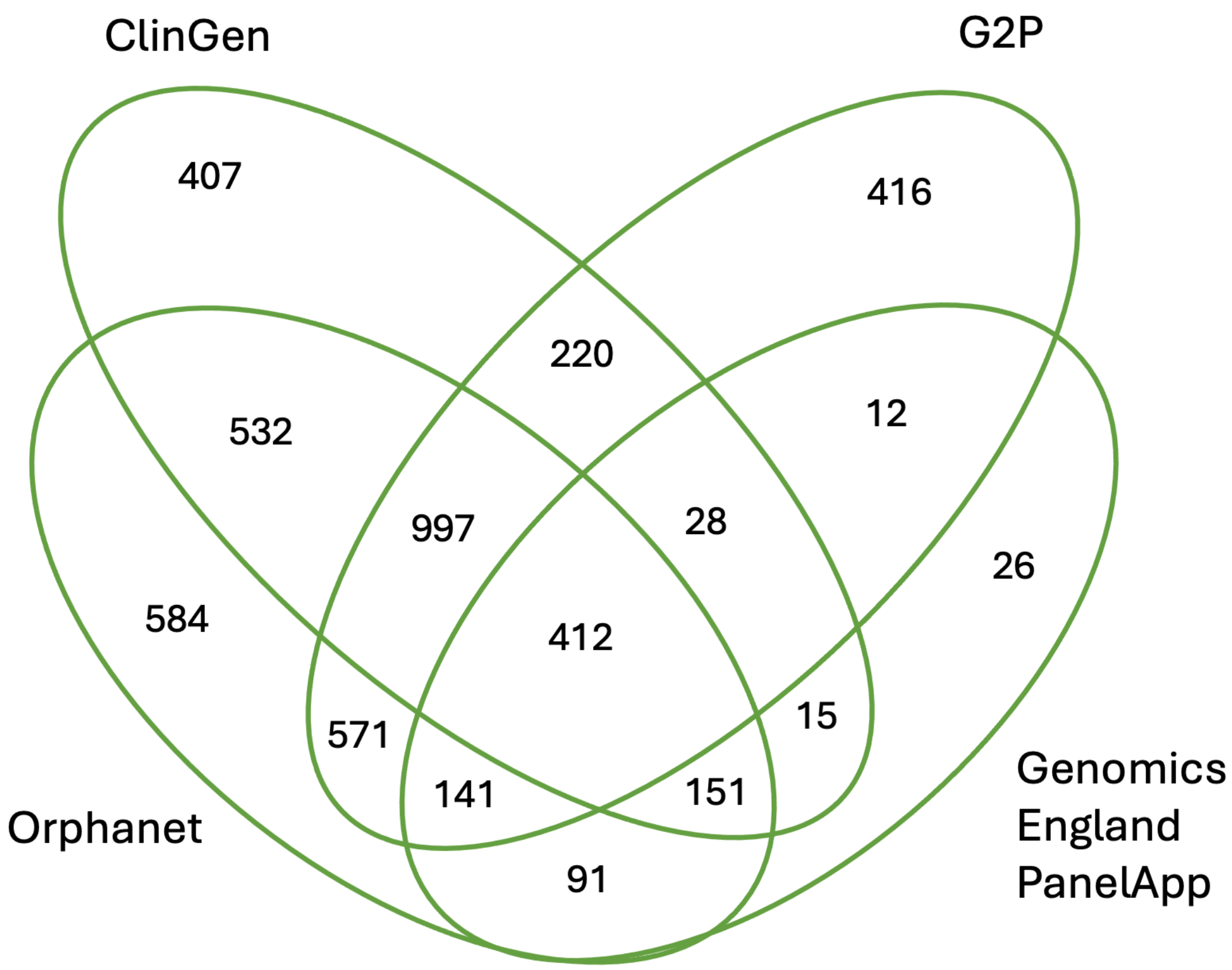

Supplementary Figure 9. Counts of genes for which at least 1 record was submitted to GenCC by 4 open gene disease curation resources.

OMIM does not currently submit data to GenCC, but 80% of G2P records have been linked to an OMIM disease identifier by curators. Of those without, 52% are from panels which have not seen a concerted effort to curate these cross references. 43% of the remaining records are of limited confidence, for which the literature is being monitored (for example ACTG1-related isolated ocular coloboma). The remaining set includes records with moderate support which are not yet present in OMIM (for example ‘YWHAE-related developmental delay, seizures, hypotonia and brain abnormalities’) and records where the gene is present in both resources but the associated disorder is described differently (for example GABRD, which is linked to ‘GABRD-related neurodevelopmental disorder with

epilepsy' in G2P and 'Generalized epilepsy with febrile seizures plus, type 5, susceptibility to' in OMIM.

The number of records contributed by each submitter were also counted and the use of standard confidence terms for gene disease association validity and the submission of PMIDs as evidence, which enhance reusability was flagged (Supplementary Table 1).

**Submitter (of > 500 records)**	**Records submitted**	**Confidence assertion**	**Evidence provided (publications)**
PanelApp Australia	1,043	Yes	96%
Genomics England PanelApp	1,250	Yes	8%
G2P	3,341	Yes	90%
ClinGen	3,468	Yes	2%
Ambry Genetics	4,216	Yes	0
Orphanet	5,401	No	93%
Labcorp Genetics (formerly Invitae)	5,528	Yes	99%

Supplementary Table 1: Counts of records submitted to GenCC and percentage with evidence provided to enable checking of conclusions. Only groups contributing more than 500 records are listed. GenCC submission information was downloaded on 2026-02-08 from https://search.thegencc.org/download.

Supplementary Methods

Implementation

The G2P system is implemented in Python and JavaScript using a custom MySQL database for data storage. All code is open source and available on GitHub (https://github.com/EBI-G2P/gene2phenotype_api, https://github.com/EBI-G2P/gene2phenotype_web).

The Web API (https://www.ebi.ac.uk/gene2phenotype/api/) was developed using the Django REST Framework to allow programmatic access to the G2P curated data. The API documentation is created using DRF Spectacular, a tool that works with the Django REST Framework to automatically generate a standardised description of the API using the OpenAPI format. This documentation explains how to interact with the G2P API, how data can be retrieved and also includes detailed descriptions of the format of the returned data. Providing standard documentation improves transparency and enables integration into other tools and workflows.

The frontend of the Website is built using Vue.js, a modern framework that enables the development of a user-friendly interface for browsing data. Security and access control are also essential to the implementation. Only registered users can access the curation interface

and they can update only the panel(s) to which they contribute. When logged in the curator can view additional records they have been working on in draft. There is also the option to start building a panel privately to release when there are sufficient records for it to be of use. Authentication is managed through Simple JWT, a secure and widely used token-based authentication system compatible with the Django REST Framework. All actions performed by curators are recorded, including the creation and deletion of records to provide a history of all changes made to a record over time.

Data curation and validation

Scientific data curation is a labour-intensive process, and errors in data extraction can render results useless or misleading. To reduce errors and support a more efficient curation process, we developed an improved web-based curation interface. G2P curators are guided through a standardised protocol using a custom data input form which performs quality assurance checks and prevents the erroneous creation of duplicate entries. Controlled vocabularies are used throughout for core attributes, with free text fields to capture additional descriptions of non-standard features.

G2P records are curated by teams composed of clinical scientists, practising clinicians and scientific curators. Different team members have different data publishing and update privileges according to experience level. Individuals curate papers and create draft G2P records which capture the reported evidence and the curators recommendations for confidence level, mechanism and disease name in a structured format. Draft records are presented in curation team meetings, evidence reviewed and aspects such as mechanism, confidence and disease name are discussed and agreed.

Initially, a gene is entered and the user is prompted for the publication(s) they are curating. When a PubMed identifier is entered, the title is retrieved, using the EuropePMC API, and displayed so input errors are immediately caught. The phenotypes reported in a publication are entered by typing the first characters of the description, triggering a look up via the HPO API (https://ontology.jax.org/api/hp/docs) so the established term is used, improving interoperability. The user is guided through the entry of required fields, selecting from controlled vocabularies, which use HPO and SO terms where available. Specific variants described in HGVS format can be optionally entered, to report recurrent variants for use in diagnostic variant filtering.
The types of evidence supporting the inferred mechanism can be selected from a predefined in list, taken from the ClinGen Gene-Disease Validity Curation Process standard operating procedure (https://clinicalgenome.org/docs/gene-disease-validity-standard-operating-procedures-version-10/) to enable interoperability between resources. Where the reported evidence provides support, more detailed molecular mechanism terms are selected from predefined lists, derived from Backwell and Marsh [5] (Supplementary Figure 10). Predictions of likely gene disease mechanism [31] and tolerance to loss of function [32, 33] are displayed to aid evaluation of the evidence of mechanism.

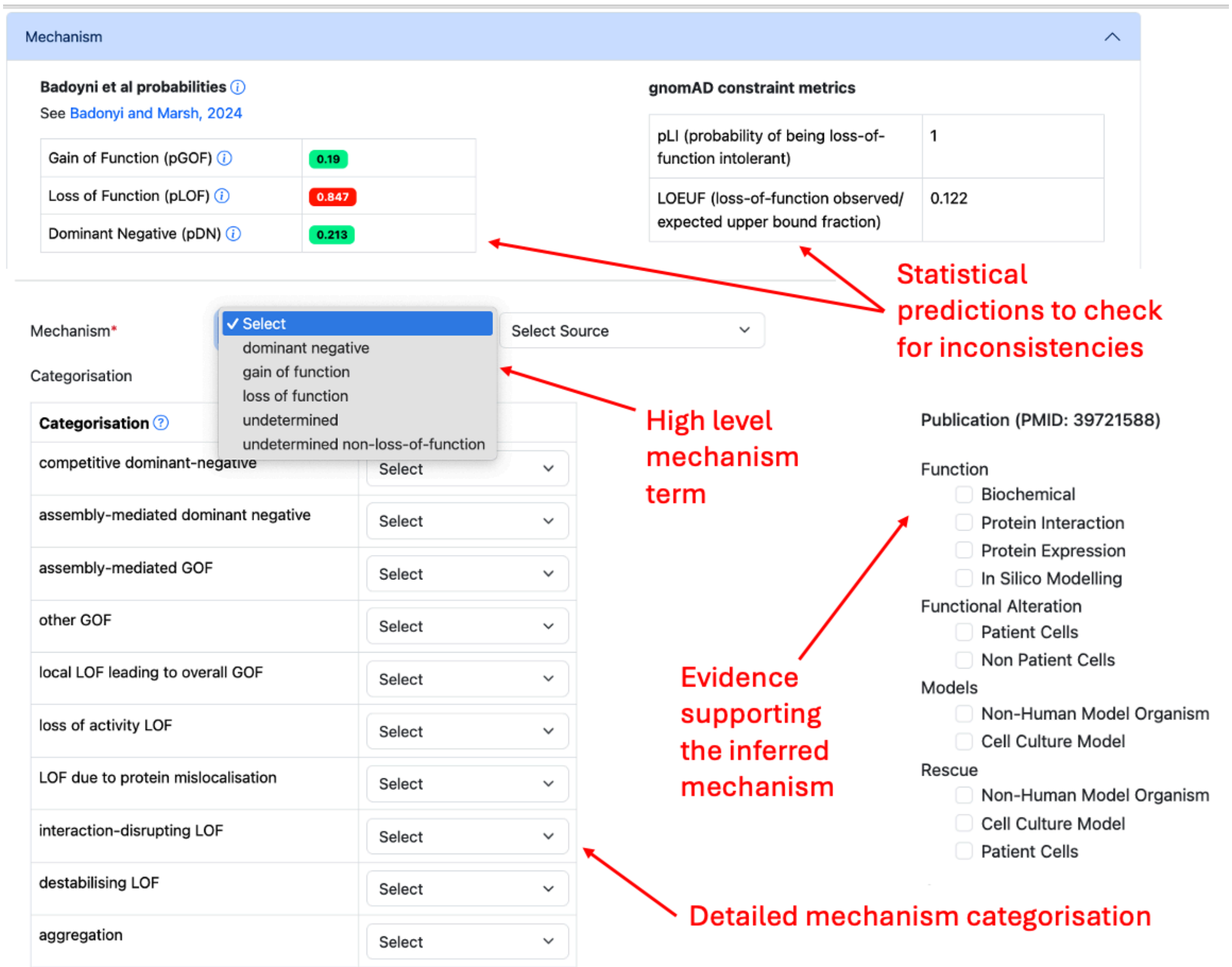

Supplementary Figure 10. Elements of the G2P curation interface supporting curation of mechanisms. Evidence types are entered with reference to the publication reporting them while overlap mechanism terms are assigned based on the evidence from all curated publications.

When deciding the confidence of the disease association, the curator is presented with a summary of the evidence they have entered and links to other resources which may have assessed the gene. To improve interoperability, the curator is presented with diseases reported to be associated with the gene by OMIM and Mondo and prompted to use these as the basis of the G2P disease name. The curator can also approve or reject suggested links to disease records in these resources.

The G2P curation interface is designed to ensure records conform to the data model and to enforce biological constraints. Validation checks run on input include:

- Allelic requirement must be compatible with the genomic location of the gene. For example, an allelic requirement of “monoallelic_X”, can only be entered for genes located on the X chromosome.
- The number of individuals reported as being affected with a disorder in a publication cannot be smaller than the number of families input.
- Where mechanism categorisations are entered, they must be consistent with the high-level term. For example, having selected ‘loss of function’ as the mechanism, ‘assembly-mediated dominant negative’ cannot be chosen as the more detailed term.

- Variant consequence terms should be feasible given the variant types observed in the patients. For example, inferring a consequence of 'altered gene product structure' for a variant of type 'regulatory region variant' reported in the publication will be flagged as an error.
- Confidence assignment must not violate the agreed definition, for example an assertion cannot be labelled as the highest confidence, definitive, if only one publication has been entered to support it, as this requires at least 4 publications.

Reference data is imported using custom Python scripts (https://github.com/EBI-G2P/gene2phenotype_scripts) and used to guide and to check user input. Diseases already reported to be associated with a gene are suggested as cross-references for the curator to select, Protein function descriptions from UniProt and predictions of likely gene disease mechanism and loss of function constraint are displayed aid the curator in assessing the published information.
Work is ongoing to update older G2P records to the new curation protocol and integrity checks are routinely run to report missingness levels, detect data inconsistencies and potential errors and prioritise entries for review.

The validation checks performed at submission ensure the new entry complies with the data model and meets defined standards. The new interface, with controlled data input and auto-complete options, along with the review steps employed in the curation process enables the capture and dissemination of high-quality and consistently described models of monogenic disease through G2P.

Additional references

[31] M. Badonyi, J.A. Marsh, Proteome-scale prediction of molecular mechanisms underlying dominant genetic diseases, PLoS One 19 (2024) e0307312. https://doi.org/10.1371/journal.pone.0307312

[32] K.J. Karczewski, et. al., The mutational constraint spectrum quantified from variation in 141,456 humans, Nature 581 (2020) 434–443. https://doi.org/10.1038/s41586-020-2308-7.

[33] M. Lek, et. al., Analysis of protein-coding genetic variation in 60,706 humans, Nature 536 (2016) 285–291. https://doi.org/10.1038/nature19057.
